# Mitochondria May Mediate Prenatal Environmental Influences in Autism Spectrum Disorder

**DOI:** 10.3390/jpm11030218

**Published:** 2021-03-18

**Authors:** Richard E. Frye, Janet Cakir, Shannon Rose, Raymond F. Palmer, Christine Austin, Paul Curtin, Manish Arora

**Affiliations:** 1 Barrow Neurological Institute at Phoenix Children’s Hospital, Phoenix, AZ 85016, USA; 2 Department of Applied Ecology, North Carolina State University, Raleigh, NC 27695, USA; Janet_Cakir@nps.gov; 3 Department of Pediatrics, Arkansas Children’s Research Institute, University of Arkansas for Medical Sciences, Little Rock, AR 72202, USA; SROSE@uams.edu; 4 Department of Family and Community Medicine, University of Texas Health Science Center, San Antonio, TX 78229, USA; palmerr@uthscsa.edu; 5 Department of Environmental Medicine and Public Health, Icahn School of Medicine at Mount Sinai, New York, NY 10029, USA; christine.autsin@mssm.edu (C.A.); paul.curtin@mssm.edu (P.C.); manish.arora@mssm.edu (M.A.)

**Keywords:** autism spectrum disorder, mitochondria, oxidative stress, prenatal environment, immune dysfunction

## Abstract

We propose that the mitochondrion, an essential cellular organelle, mediates the long-term prenatal environmental effects of disease in autism spectrum disorder (ASD). Many prenatal environmental factors which increase the risk of developing ASD influence mitochondria physiology, including toxicant exposures, immune activation, and nutritional factors. Unique types of mitochondrial dysfunction have been associated with ASD and recent studies have linked prenatal environmental exposures to long-term changes in mitochondrial physiology in children with ASD. A better understanding of the role of the mitochondria in the etiology of ASD can lead to targeted therapeutics and strategies to potentially prevent the development of ASD.

## 1. Introduction

Autism spectrum disorder (ASD) is a behaviorally defined disorder [1], with the most recent Center for Disease Control and Prevention estimates suggesting that it affects 1 in 54 children in the United States [2]. Recent studies suggest that inherited single-gene and chromosomal defects account for a minority of ASD cases [3], and that ASD most likely arises from a complicated interaction between genetic predisposition and environmental exposures [4,5]. Given the high recurrent risk in siblings, the prenatal maternal environment has undergone careful study with many prenatal risk factors identified [6,7]. Despite the epidemiological connection between many prenatal risk factors and the development of ASD, the biological mechanisms which link prenatal environmental influences and the increased risk of developing ASD are just beginning to be uncovered. 

Three physiological abnormalities which have been increasingly recognized to be associated with ASD are immune system dysfunction, mitochondrial dysfunction, and oxidative stress and redox regulation [1]. Previous reviews examining prenatal physiological abnormalities related to ASD have concentrated on prenatal immune stressors as key and consider mitochondrial dysfunction and oxidative stress to have secondary roles of this “Bad Trio” [8]. In contrast, the current review concentrates on the mitochondria as the central player. Of course, the particular component of the “Bad Trio” that is the initiating culprit may be different for different patients and it is possible that multiple stressors on the various portions of the “Bad Trio” simultaneously may also initiate the pathway to disease. 

## 2. The Mitochondria: Dysfunction Can Be Self-Perpetuating

**Figure 1:**
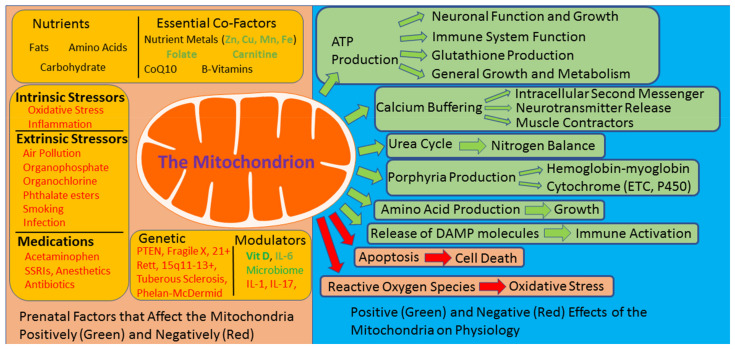
The mitochondria can be negatively affected by many environmental and biological factors associated with autism spectrum disorder (left orange panel) and has many critical roles in cellular physiology (right blue panel). ETC: electron transport chain; DAMP: damage-associated molecular pattern

Mitochondria are essential for a wide range of functions in almost every cell in our body (Figure 1). Best known for their role in the production of adenosine triphosphate (ATP) by oxidative phosphorylation, mitochondria are intimately involved in other cellular functions such as redox metabolism, calcium buffering, lipid homeostasis, and steroid synthesis [9,10,11,12,13]. Mitochondria also have a role in important non-energy-producing metabolic pathways, such as the urea cycle, amino acid and porphyrin production, and as a pathway for the activation of apoptosis. Mitochondria also have important roles in cell signaling, most notably being an essential part of the inflammasome, a complex that initiates immune activation, by releasing damage-associated molecular pattern (DAMP) molecules such as cardiolipin, n-formyl peptides, reactive oxygen species (ROS), and mitochondrial DNA (mtDNA) [14]. Lastly, normal mitochondrial function results in the production of ROS, which can cause cellular injury if not controlled.

**Figure 2:**
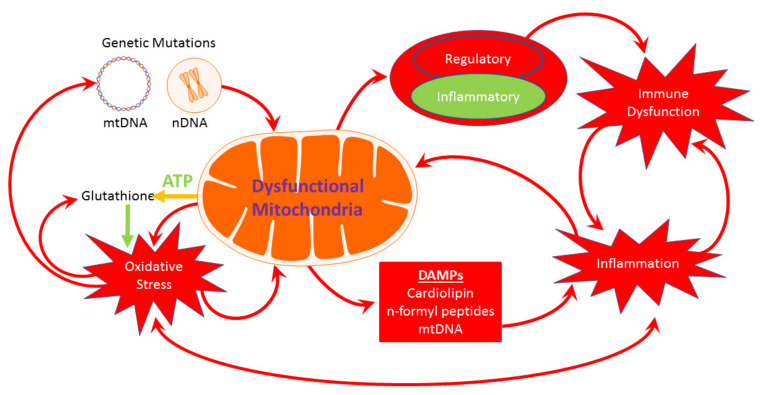
Self-perpetuating destructive cycles which can result in mitochondrial dysfunction.

Since ATP produced by mitochondria is essential for many cellular systems, abnormal mitochondrial function can disproportionally adversely affect cellular physiology. However, there are several pathways in which abnormal mitochondrial function can result in a self-perpetuating destructive cycle causing sustained pathophysiology. Most notably, interactions between mitochondria, redox metabolism, and the immune system can be mutually detrimental; such detrimental interactions have been documented in ASD (Figure 2) [15].

Mitochondria are both a major producer and target of ROS. Dysfunctional mitochondria produce high amounts of ROS which can result in dysfunction of the electron transport chain (ETC) enzymes, particularly complex I and III, as well as aconitase, the first enzyme in the citric acid cycle (CAC). To compound this problem, reduced glutathione (GSH), the main intracellular and mitochondrial antioxidant, requires ATP for its *de novo* production. As such, a decrease in ATP production resulting from reduced mitochondrial function will result in lower GSH production, resulting in poorer control of ROS. In fact, a lower GSH redox ratio has been correlated with lower aconitase activity in post-mortem brain from individuals with ASD [16]. Oxidative damage to cellular lipids, proteins, and nucleic acids [17] has been associated with ASD; this is especially important since mtDNA is vulnerable to oxidative damage, and studies have shown that children with ASD demonstrated mtDNA damage in a pattern consistent with oxidative damage [18].

There are several pathways in which dysfunctional mitochondria can cause a wide variety of abnormalities in immune system function. First, cellular damage due to oxidative stress can activate inflammatory pathways [15,16]. Second, as an essential part of the inflammasome, mitochondria release DAMP molecules such as cardiolipin, n-formyl peptides, ROS, and mtDNA [14]. Third, regulatory immune cells are highly dependent on ATP derived from mitochondrial oxidative phosphorylation, while inflammatory cells are highly glycolytic [19]. Thus, once an immune response has been started, the inflammatory response may be difficult to regulate if mitochondrial dysfunction exists. Fourth, inflammation and immune activation upregulate metabolism and recruit physiological processes, but without mitochondrial support, such resources will not be available. Lastly, the immune system produces ROS as a defense mechanism against potential invaders. Such increases in ROS can result in a further detrimental effect on already dysfunctional mitochondria. In fact, mitochondria appear to have a particularly important role in innate immunity [20], which is an area of immune dysfunction that is implicated in ASD. In addition, an increase in proinflammatory cytokine production has been associated with mitochondrial dysfunction in a subset of children with ASD [21]. 

## 3. Prenatal Risk Factors for ASD Modulate Mitochondrial Function

Many prenatal factors associated with an increased risk of ASD are associated with mitochondrial dysfunction; these include nutritional agents, both intrinsic and extrinsic stressors, common medications given during pregnancy, modulators of mitochondrial function, and genetic conditions which might affect the fetus (Figure 1).

Folate is an essential vitamin which is well-known to be important during pregnancy to prevent neural tube defects. Folate is also an essential co-factor for adequate mitochondrial function [22]. Several studies have demonstrated that folate supplementation during pregnancy reduces the risk of ASD. Abnormalities in the folate pathway are associated with ASD, including maternal polymorphisms in the reduced folate carrier [23]. Further, mothers of children with ASD have been shown to have the folate receptor alpha autoantibody [24], an abnormality that prevents folate transport across the placenta, resulting in an ASD phenotype in an animal model [25].

Abnormalities in several nutrient metals have been linked to an increased risk of ASD. Studies have associated an increased risk of ASD with atypical pre- and postnatal Zn and Cu metabolism [26] and found that atypical levels of essential (Zn, Mn) and non-essential (Pb) metals during prenatal development and early life in individuals with ASD are associated with long-term physiological and developmental alternations [27]. Studies have associated low maternal iron (Fe) with increased ASD risk [28]. Prenatal Zn and Cu are essential for the function of the cytoplasmic superoxide dismutase (SOD) which is essential for controlling cellular oxidative stress, while Cu is essential for ETC complex IV function. Mn is essential for mitochondrial SOD function, and Fe is an essential component of cytochromes, which are critical components of the ETC.

Carnitine is an essential nutrient for mitochondrial function and fatty acid metabolism [11,29,30]. Abnormalities in carnitine metabolism have been linked to gestational diabetes [31], a risk factor for ASD [32]. A genetic defect in carnitine synthesis is a risk factor for ASD [33], carnitine metabolism is known to be disrupted in ASD [29,30] and a mouse model of ASD is associated with alternations in carnitine biosynthesis [34]. Due to its importance in energy metabolism in neural stem cells of the developing mammalian brain, carnitine deficiency has been proposed to be a prenatal risk factor for ASD [35].

As noted above, increased oxidative stress as well as inflammation is associated with mitochondrial dysfunction. Abnormalities in maternal trans-sulfuration metabolism and chronic oxidative stress are found in mothers of children with ASD during [36] and following [37] pregnancy and, in general, infection during pregnancy is a risk factor for ASD [38]. In fact, the maternal immune activation (MIA) mouse model of ASD demonstrates long-term mitochondrial dysfunction in brain [39] and leukocytes [40] after birth. Pregnancies resulting in a child with ASD have demonstrated increases in proinflammatory cytokines IL-1α [41] and IL-6 [41] in blood, while maternal elevation in IL-17a has been strongly implicated in the MIA model [42]. In laboratory studies, IL-17 [43,44] induces mitochondrial dysfunction through activation of the mitochondria-induced apoptosis pathway and IL-1 suppresses mitochondrial function [45], while IL-6 promotes mitochondrial biogenesis and fatty acid oxidation [46].

Prenatal exposure to many environmental toxicants is linked to an increased risk of developing ASD, including cigarette smoke, phthalates, air pollution, and pesticides such as organophosphate insecticides (e.g., chlorpyrifos) and organochlorine pesticides (e.g., dicofol and endosulfan) [7]. These toxicants have been associated with mitochondrial dysfunction. Organophosphate induces mitochondrial ultrastructure changes and inhibits ETC and CAC enzyme function, while organochlorine pesticides influence mitochondrial dysfunction indirectly by increasing ROS and reactive nitrogen species by altering antioxidant systems including SOD and GSH [47]. Phthalates, common plasticizers present in everyday products, have also been linked to increased oxygen consumption, mitochondrial mass, and fatty-acid metabolism in neonatal rat cardiomyocytes [48] and detrimental changes in mitochondrial membrane potential in human semen [49]. Mitochondrial-derived peptides in cord blood have been associated with prenatal exposure to non-freeway traffic-related air pollution [50]. Cigarette smoke causes mitochondrial dysfunction in the lung epithelium [51].

Several medications commonly used during pregnancy have been linked to an increased risk of developing ASD including acetaminophen [52] and selective serotonin reuptake inhibitors [53]. Acetaminophen has known toxicity by increasing reactive metabolites leading to suppression of mitochondrial function [54]. Fluoxetine, a commonly used selective serotonin reuptake inhibitor, has been shown to inhibit multiple mitochondrial enzymes in several laboratory studies [55] and may result in long-term changes in energy metabolism with neonatal exposure [56]. Other medications commonly used in pregnancy with less certain association with ASD are associated with mitochondrial dysfunction. For example, commonly used antibiotics, including quinolones, aminoglycosides, and β-lactams, can cause mitochondrial dysfunction [57], and commonly used anesthesia may be particularly detrimental to the developing brain, partially through mitochondrial mechanisms [58]. 

Interestingly, mitochondrial abnormalities have been documented in genetic syndromes associated with ASD such as PTEN mutations [59] and tuberous sclerosis [60,61], Fragile X [62,63], Rett [64,65,66], Phelan–McDermid [67], 15q11-13 duplication [68,69], Angelman [70] and Down [71,72] syndromes as well as septo-optic dysplasia [73]. Thus, a fetus with these genetic changes may already have vulnerable mitochondria that might be sensitive to environmental stressors.

Common nutritional deficiencies are prenatal ASD risk factors which have been suggested to modulate mitochondrial function. Decreased vitamin D in the first [74] or second [75] trimester as well as lifetime [76] is associated with more severe ASD [74,75] or increased ASD risk [76]. Vitamin D deficiency is associated with oxidative stress and reduced mitochondrial respiration that is mediated through the vitamin D receptor [77,78]. 

Several lines of evidence suggest that ASD is associated with disruption of the microbiome in individuals with ASD as well as their mother during pregnancy [79]. Although a recent landmark study provided preliminary evidence that transplanting the microbiome in individuals with ASD can improve gastrointestinal and ASD symptoms [63], studies in pregnant women are more difficult to conduct, leading to animal studies addressing this possibility. Environmentally induced rodent models of ASD, including the MIA [80] and the valproic acid exposure models [81,82,83], have an altered microbiome. Manipulations which can affect the microbiome have been shown to mitigate the effects of these maternal exposures. Treating pups born from maternal valproic acid exposure demonstrate reduced ASD-like behaviors [84,85] as well as normalization of mitochondrial abnormalities [84], while treatment of pups born from MIA with *Bacteroides fragilis* normalizes gut permeability and microbial composition and reduces ASD-like behaviors [86]. Interestingly, several prenatal environmental exposures linked to ASD, including air pollution [87], glyphosate [88], prenatal antibiotic use [89], maternal stress [90] and organophosphate herbicides [91], have evidence for disrupting the microbiome, suggesting a potential biological pathway for their effects. The microbiome can influence mitochondrial function through several mechanisms, although the most compelling is through the production of short chain fatty acids [92].

## 4. Unique Abnormalities in Mitochondrial Function Are Prevalent in ASD

The possibility that environmentally induced mitochondrial dysfunction could have a role in ASD is particularly compelling because abnormal mitochondrial function is one of the most prevalent metabolic disorders found in individuals with ASD, with prevalence ranging from 5% for classically defined mitochondrial disease to 8–47% for biomarkers of mitochondrial dysfunction [11,16], to 62–65% for abnormal ETC/CAC enzymology [93,94], and to 80% for abnormal ETC activity in lymphocytes and granulocytes [95,96]. Particularly compelling is that the great majority of the time, genetic defects cannot explain the mitochondrial abnormalities, suggesting that the abnormality could be acquired as a result of an environmental exposure. There is also a tremendous practical appeal in this hypothesis, as environmental determinants may be particularly amenable to modification. 

In classic mitochondrial disease, ETC activity is, by definition, depressed. However, what is unique about abnormalities in mitochondrial function in individuals with ASD is that ETC activity is significantly increased in many cases. The first case reported was a boy with ASD who demonstrated a significant increase in ETC complex I activity while his sister, who was diagnosed with a classic mitochondrial disease known as Leigh syndrome, showed depressed ETC activity [97]. Interestingly, both siblings manifested the same mtDNA mutation, but the sister had a greater genetic mutational load (i.e., higher heteroplasmy). Subsequently, a case-series of five patents with ASD with muscle ETC complex IV activity about 200% of normal was reported [98]. The association of elevated ETC complex IV activity with ASD has subsequently been confirmed in fresh frozen superior temporal gyrus [99], buccal swabs enzymology [93] and lymphoblastoid cell lines (LCLs) using high-resolution respirometry [100].

## 5. The Significance of Mitochondrial Dysfunction in ASD: Sensitivity to Physiological Stress

Since increased ROS is a key mechanism by which environmental stressors, such as toxicants [101,102,103,104,105,106,107,108] and inflammation [1,11], can disrupt mitochondrial function, an assay has been developed which systematically increases ROS *in vitro* [109]. This assay, called the Mitochondrial Oxidative Stress Test (MOST), systematically increases ROS *in vitro* using 2,3–dimethoxy–1,4-napthoquinone (DMNQ), an agent that generates intracellular superoxide and hydrogen peroxide but does not directly deplete thiols [110]. The model was initially developed using LCLs from boys with ASD and age-matched healthy controls (CNT) where DMNQ was shown to increase ROS in CNT and ASD LCLs [110].

In this model, mitochondrial respiratory rates were significantly higher in ASD LCLs as compared to CNT LCLs. Most compelling, indices of mitochondrial health, reserve capacity and maximal respiratory rate, decreased to a greater extent in ASD LCLs when challenged with DMNQ in the MOST assay. Increases in ROS, using the MOST assay, resulted in a depletion of these respiratory parameters at lower DMNQ concentrations in the ASD LCLs, despite these parameters being higher at baseline. This suggests that the ASD LCLs demonstrated a greater vulnerability to ROS.

To determine if these bioenergetics changes were specific to a subset of ASD LCLs, a cluster analysis was used to separated ASD LCLs into those with normal bioenergetics (AD-N) and those with atypical bioenergetics (AD-A) [109]. When the AD-A LCLs were compared to the CNT LCLs, the bioenergetic differences were found to be large. Baseline respiratory rates were ~200% higher in AD-A as compared to the CNT LCLs. Most notably, maximal respiratory capacity and reserve capacity markedly decreased as DMNQ increased, such that reserve capacity was rapidly depleted as DMNQ increased despite being much higher at baseline. This pattern of abnormal respiration in this subset of LCLs has been confirmed over eight studies [61,109,111,112,113,114,115,116].

Despite these experiments, the question remained whether this pattern of mitochondrial dysfunction was specific to ASD or simply a consequence of higher levels of chronic ongoing intrinsic oxidative stress. Since neurotypical siblings (SIBs) of children with ASD manifest similar chronic ongoing elevation in oxidative stress in their LCLs, mitochondrial function was compared between 10 LCLs from boys with ASD and their 10 male SIBs and 10 age-matched CNT males [117]. Mitochondrial function was similar between SIBs and CNTs, but both were different from ASD. In the ASD LCLs, mitochondrial respiration was elevated at baseline and reserve capacity declined more precipitously with increasing DMNQ, as compared to SIB and CNT LCLs. Most notably, the severity of mitochondrial abnormalities in ASD LCLs was related to the severity of stereotyped behaviors and restricted interests as measured on the gold-standard Autism Diagnostic Observation Schedule evaluation years earlier when the blood samples were original collected. Thus, atypical mitochondrial activity in the ASD LCLs is not simply a product of abnormal redox metabolism but rather associated with atypical mitochondrial function specifically. Furthermore, this atypical mitochondrial function is related to more severe core ASD behaviors, suggesting an association with molecular mechanisms of ASD.

Individuals with ASD demonstrate three developmental trajectories: in the early onset subtype, symptoms are obvious from early in infancy, perhaps at birth; in the plateau subtype, infants develop normally throughout the first year of life but then plateau in the rate of gaining skills, followed by the development of ASD symptoms; lastly is a subset that demonstrates neurodevelopmental regression (NDR). The NDR category is intriguing: children attain all their normal developmental milestones but then lose previously attained skills followed by the development of ASD-like behaviors. Often, NDR is associated with a trigger such as seizure [118] and/or fever [119]. NDR is not uncommon in individuals with mitochondrial disease when an illness occurs [120]. Thus, it is not surprising that a meta-analysis showed that NDR was more common in children with ASD that were also diagnosed with mitochondrial disease [11].

Given that the subset of ASD LCLs with elevated respiratory rates have increased vulnerability to physiological stress, a recent study hypothesized that children with ASD and NDR would demonstrate increase mitochondrial respiratory rates. Mitochondrial function was measured in cryopreserved peripheral blood mononuclear cells (PBMCs) from children with ASD, with and without NDR, as well as CNT using the Seahorse XF96 respirometer [121]. Viability was measured and 600 k viable PBMCs were plated per well. As hypothesized, mitochondrial respiration was elevated in children with ASD and NDR. Specifically, the maximal oxygen consumption rate, maximal respiratory capacity, and reserve capacity were higher in the individuals with ASD and NDR as compared to the other groups. Additionally, comparing ASD twins discordant on NDR demonstrated that the twin with NDR showed a significantly elevated maximal oxygen consumption rate. Thus, the NDR ASD phenotype may be a hallmark of abnormal mitochondrial physiology.

## 6. Unique ASD Mitochondrial Abnormalities May Be Linked to Both Environmental and Genetic Factors

Interestingly, the unique elevations in mitochondrial respiration reported in human tissue have been associated with both genetic and environmentally induced animal models of ASD. Elevated mitochondrial respiration has been documented in genetic syndromes associated with ASD, including Phelan–McDermid [122], Fragile X [62,63], 22q13 dup [67], and Rett [66] syndromes, the PTEN haploinsufficient mouse model of ASD [59] and the Drosophila model of the ASD associated CYFIP1 mutation [123]. Similar changes have been associated with prenatal environmental exposures, specifically prenatal exposure to inflammation as in the MIA mouse, a model of ASD induced by prenatal immune environmental stress [39], and prenatal exposure to toxins as in the maternal valproic acid exposure mouse model of ASD [84].

Consistent with evidence from these prenatal environmental animal models of ASD, several studies have examined the relationship between alternations in long-term mitochondrial function and prenatal environmental stressors in ASD.

Prenatal exposure to air pollution, as measured by average and maximum PM_2.5_, has been found to be related to mitochondrial respiration in childhood, as measured in PBMCs. The relationship was significantly different for those children with and without a history of NDR. For those with a history of NDR, higher prenatal PM_2.5_ exposure was associated with higher mitochondrial respiration rates, while for those without a history of NDR, higher prenatal PM_2.5_ exposure was related to lower mitochondrial respiration [27]. Additional research has linked prenatal air pollution exposure to mitochondrial-derived peptides in cord blood that are associated with long-term changes in mitochondrial physiology [50].

Both prenatal exposures to nutritional and toxic metal were measured in deciduous teeth using laser ablation inductively coupled plasma mass-spectrometry. Prenatal exposure to Mn and Zn was associated with mitochondrial respiration, but only in children with ASD and NDR [29]. The prenatal Cu to Zn ratio was associated with two independent measures of language development in all children with ASD (both those with and without NDR) [29]. This latter study extends previous findings linking prenatal nutrient metal (Zn, Mn, Cu) exposure and ASD [26,27,124,125,126,127]. 

To determine whether the long-term changes associated with ASD could be induced by exposure to environmental toxicants, one study exposed LCLs to low levels of ROS for a prolonged time (96 h) to simulate chronic ROS exposure which might occur with prolonged exposure to environmental toxicants or other physiological stressors [61]. Prolonged exposure to low levels of ROS induced bioenergetic changes in respiratory parameters involved in ATP production. After prolonged ROS exposure, the LCLs demonstrated increased respiratory rates at baseline, similar to the AD–A LCLs and the ETC activity seen in children with ASD in multiple tissues. These data suggest that a prolonged exposure to a pro-oxidant microenvironment can have chronic effects on the mitochondria.

## 7. Long-Term Induced Changes in Mitochondrial Function: Adaptive or Maladaptive

Mitochondria undergo long-term adaptive changes in physiology as a result of environmental stressors through a process known as mitoplasticity, which is one compelling mechanism for long-term changes in mitochondrial function associated with prenatal exposures [9]. Several studies may provide insight into the molecular mechanisms associated with long-term changes in mitochondrial function.

Matched typically developing siblings have similar abnormalities in oxidative stress compared to their ASD siblings, but the mitochondria of the ASD siblings appear to have difficulty regulating the mitochondrial ROS (mtROS). mtROS is regulated at the inner mitochondrial membrane through several mechanisms. Uncoupling proteins regulate mtROS by leaking protons across the inner mitochondrial membrane, a process known as proton leak. Studies have demonstrated increased proton leak respiration along with high oxidative stress in both cytoplasm and mitochondria in ASD LCLs [16,128]. Studies have associated ASD with an increase in mechanisms of proton leak. Three previous studies have found an increase in uncoupling protein (UCP2) gene expression [61,116] and protein concentration [109] in LCLs derived from children with ASD, particularly in the LCLs with high respiratory rates (i.e., AD–A LCLs). The adenine nucleotide translocator (ANT) has a significant role in the regulation of inner mitochondrial membrane proton leak. Microdeletion including the SLC25A5 (ANT2) has been associated with non-syndromic intellectual disability with ASD [129]. Heteroplasmic levels of the mtDNA 3243A > G mutation associated with ASD are also associated with significant changes in ANT gene expression [130]. Consistent with this evidence, increased protein leak is also a feature of mitochondrial abnormalities in the Fragile X syndrome mouse, where it is found to directly affect synaptic growth [131]. Thus, dysregulation in inner membrane proton leak seems to be associated with an ASD phenotype.

One of the key processes which maintains optimal mitochondrial function in the face of physiological stress is mitophagy. Several studies have linked ASD to a failure to induce this important process. Mutations in WDFY3 which result in ASD with intellectual disability have been linked to bioenergetic abnormalities in the brain through decreased mitophagy [132], and PARK2, a gene known to be involved in mitophagy, has been identified as a candidate gene for ASD [133]. Examination of the post-mortem temporal cortex has demonstrated ETC activity abnormalities along with alternation in levels of fission (Fis1, Drp1) and fusion (Mfn1, Mfn2 and Opa1) protein essential for regulating mitophagy [134]. Neurons deficient in TSC1/TSC2, a model of tuberous sclerosis, demonstrate impaired mitophagy through a mTORC1 mechanism [60,135]. This is consistent with studies which have demonstrated that ASD LCLs with elevated respiratory rates (i.e., AD–A LCLs) fail to upregulate genes associated with mitophagy through a mTORC1 pathway related mechanism [61]. Recently, ASD-derived fibroblasts were found to have elevated respiratory rates, atypical mitochondrial morphology, and alteration in the mitophagy pathway [136]. Interestingly, a loss of mitophagy can lead to a build-up of cytosolic ROS and mtDNA damage and an increase in proinflammatory cytokines, including IL–1α, IL–1β, IL–18, IFNα, MIF, IL–23, and IL–17 [137].

Alternative to regulatory changes within the body is the possibility that long-term alternation in the microbiome environment (technically outside of the body) could account for long-term changes in mitochondrial function. The connection between the microbiome and mitochondrial dysfunction in ASD is supported by the compelling propionic acid (PPA) rodent model of ASD. Adult rats intraventricularly injected with PPA [138], juvenile rates intraperitoneally injected with PPA [139], and prenatally PPA exposed rodents [140,141] demonstrated ASD-like behaviors. The importance of this model is that fact that enteric microbiome producers of PPA, particularly *Clostridia* sp, are relatively overrepresented in the ASD microbiome [142]. PPA exposure results in several physiological abnormalities, including mitochondrial dysfunction, particularly disruption of the fatty acid metabolism, as manifested by unique elevations in acyl-carnitines, as well as redox abnormalities as manifested by GSH alternations [142]. Parallel to the unique acyl-carnitine elevations in the rodent model, the same pattern was reported in a case series of ASD patients [143] with the parallel mitochondrial and GSH abnormalities later reported in a more detailed study [144]. Further *in vitro* studies with ASD LCLs demonstrated the potential detrimental effects of PPA [114] and the protective effect of another prominent enteric microbiome derived short chain fatty acid butyrate [115]. Thus, long-term changes in the microbiome could also account for long-term changes in mitochondrial function in individuals with ASD.

## 8. Conclusions

One of the critical knowledge gaps in environmental medicine is how an environmental exposure can result in disease when the symptoms arise years after the exposure. Mitochondrial metabolism is a promising mechanism through which the environment can cause long-term effects. For ASD, we have discussed the many prenatal environmental exposures which are linked to ASD and can cause detrimental changes in mitochondrial function. This is compelling because mitochondrial dysfunction is prevalent in ASD. ASD is also associated with a unique type of mitochondrial dysfunction which may be related to environmental exposures. Studies support the notion that specific prenatal exposures linked to ASD, most notably air pollution and prenatal nutritional metal exposure, is associated with long-term alterations in mitochondrial physiology. A better understanding of these biological processes may lead to prevention and treatment strategies.

Prenatal mitochondrial function is important for brain development, as recent animal studies have shown that mitochondrial dysfunction during gestation alters white matter brain connectivity [145] and non-radial interneuron migration [146]. Mitochondrial function is essential for optimal neuronal function and is essential for transport of essential nutrients into the brain such as folate [30]. Mitochondria are also essential for important core metabolic pathways as well as for optimal function of the immune system, which is now known to have an important role in brain development [30]. Thus, mitochondrial dysfunction during the prenatal period can have a profound effect on long-term development.

In this manuscript, we discuss studies with findings that link prenatal environmental exposures to long-term mitochondrial function and ASD. These studies remain intriguing yet preliminary. These findings need to be confirmed and further studies need to be conducted to expand on these findings. One important caveat is a consideration of the measurement of mitochondrial function, as mitochondrial function can differ from tissue to tissue and various assays reflect different aspects of mitochondrial function. For example, the use of PBMCs to measure mitochondrial function is a relatively newly developed biomarker which will require further validation in the future [147].

The applications of these findings may be far reaching, as interventions to correct metabolic and mitochondrial abnormalities are under development, potentially providing targeted treatments for preventing ASD and other diseases from developing if implemented prenatally.
